# Duane Retraction Syndrome: A Report of Two Cases and Review of Literature

**DOI:** 10.7759/cureus.74460

**Published:** 2024-11-25

**Authors:** Abdullah Abu Melha, Abdullah I Abbas, Wejdan S Alghamdi, Shima M Alghamdi, Abdulmajeed Alkhathami

**Affiliations:** 1 Department of Ophthalmology, King Fahad Hospital, Al Baha Health Cluster, Al Baha, SAU; 2 Faculty of Medicine, Ibn Sina College, Jeddah, SAU; 3 Department of Ophthalmology, College of Medicine, University of Bisha, Bisha, SAU

**Keywords:** abducent nerve, case report, congenital condition, saudi arabia, stilling-duane syndrome

## Abstract

Stilling-Duane syndrome, a congenital condition characterized by aberrant innervation of the lateral rectus muscle and agenesis of the abducent nerve or its nucleus, results in limited horizontal eye movements. It is often misdiagnosed as acquired abducent nerve paralysis. This report highlights the importance of considering Stilling-Duane syndrome in differential diagnoses. Case 1 is a 26-year-old female who presented to the Ophthalmology Clinic with recurrent ocular pain and puffiness over one month. She reported discomfort in both eyes, redness, and eyelid swelling but denied double vision or any significant medical history. Examination revealed normal visual acuity, marked restriction of abduction in the left eye, and asymmetry in palpebral fissure height during adduction. Horizontal nystagmus was noted at extreme abduction of the right eye. Case 2 is a 12-year-old female presented to the Ophthalmology Clinic with decreased vision in the left eye. Examination revealed decreased visual acuity in the left eye, marked restriction of abduction in the left eye, and asymmetry in palpebral fissure height during adduction. Given our patients' diagnosis of Duane retraction syndrome (DRS) type 1 with normal head posture, a management plan of observation was recommended. Surgical intervention may be considered in the future if significant changes such as abnormal head position or strabismus occur. This case emphasizes the need for careful evaluation of congenital eye movement disorders to avoid misdiagnosis and ensure appropriate management.

## Introduction

Sinclair, Turk, and Stilling provided the first description of Duane retraction syndrome (DRS) in 1895. Duane described the clinical manifestations and proposed a theory for the etiology and management of the disease in 1905 [1]. DRS is typified by restricted abduction and retraction of the afflicted eyeball; during adduction, the palpebral fissure narrows. These symptoms are frequently linked to different levels of adduction restriction with the afflicted eye moving up or down following adduction [1-3]. Congenital structural defects have been reported to be the most common cause of DRS. Among the congenital disorders of cranial dysinnervation, this is the most common type. Its prevalence is 0.1% in the general population and 1-4% in patients with strabismus. People who suffer from DRS can have either exodeviation or esodeviation. The cause of DRS is an abnormal development in specific centers of the brain stem that control the muscles outside the eye [2]. There are two theories on DRS: the most widely recognized is neurogenic, and the other is myogenic, which involves congenital fibrosis of the afflicted muscles. This idea states that the third nerve abnormally innervates the lateral rectus and that the sixth cranial nerve or its nucleus is hypoplastic [2]. It is divided into three categories. The sixth cranial nerve's hypoplastic nucleus might restrict the afflicted eye's abduction in its most prevalent form (type 1). 

People with DRS are often diagnosed in childhood and may also suffer from neck stiffness or strabismus [3,4]. In this study, we discuss two cases of Duane syndrome type 1. We hope that our cases will be a valuable addition to the existing literature.

## Case presentation

Both cases presented at the Ophthalmology Clinic in King Fahad Hospital within a span of three months. The inclusion of these cases was consecutive as they were evaluated based on their clinical presentation upon arrival. The two patients had no known relationship in common, and this is explicitly noted in the manuscript.

Case 1

**Figure 1:**
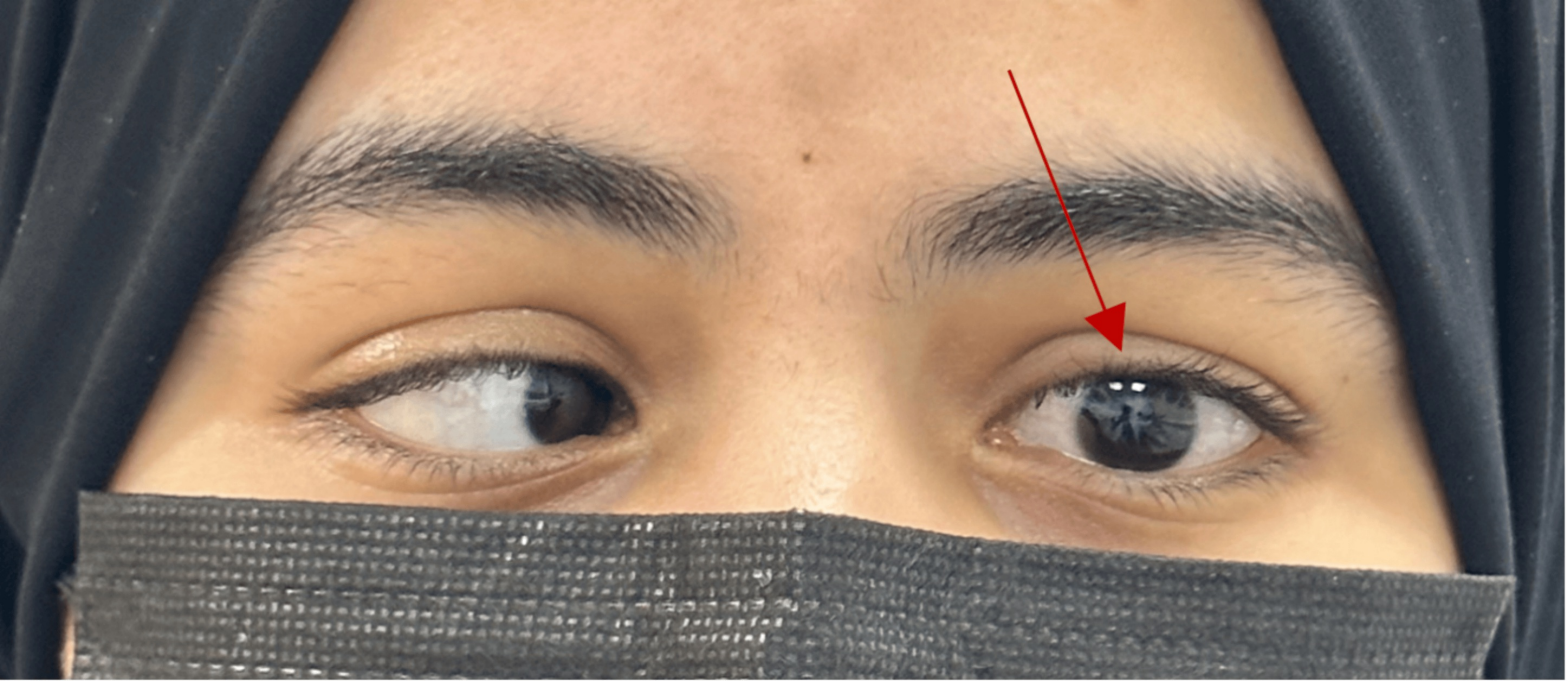
Deficit in left eye abduction

A 26-year-old female was referred to the Ophthalmology Clinic with complaints of recurrent ocular pain and puffiness for one month. The patient described episodes of discomfort in both eyes, often accompanied by redness and swelling of the eyelids. She denied any sensation of double vision and denied any history of trauma, recent infections, or allergies. The patient had no significant past medical history and had not undergone any ocular surgeries. There were no known systemic illnesses. There was no relevant family history of ocular disorders, strabismus, or hereditary eye conditions. The systemic history was unremarkable, with no reports of headaches, nausea, or systemic symptoms such as fever or weight loss. Her ophthalmological examination revealed that both of her eyes had 20/20 visual acuity. During the motility examination, the left eye's abduction was noticeably restricted (Figure 1). 

**Figure 2:**
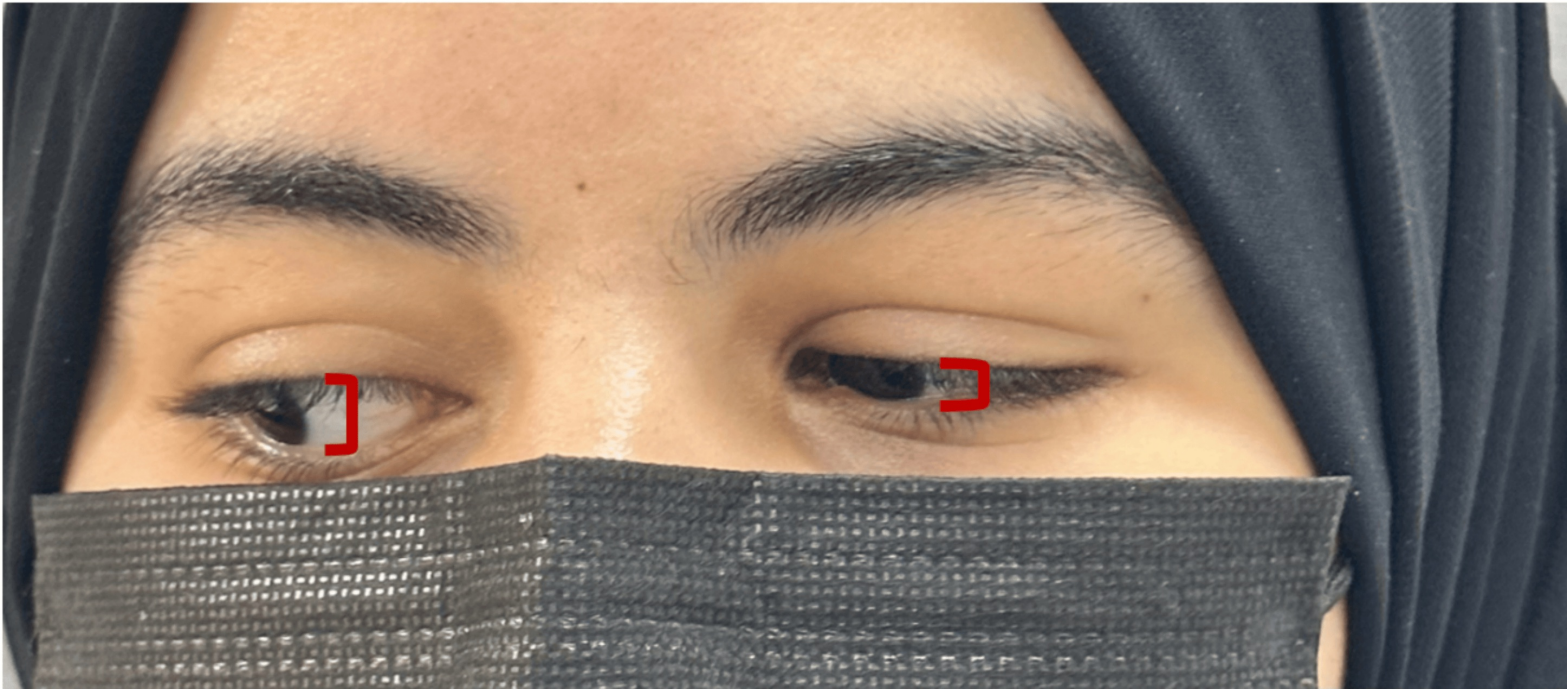
Palpebral retraction and left eye constriction upon adduction

There was limited adduction with noticeable narrowing of the palpebral fissure on adduction, particularly on the left side (Figure 2). 

Asymmetry in palpebral fissure height was noted during adduction. Horizontal nystagmus was observed in the right eye at extreme abduction (Video 1). 

**Video 1:** Horizontal nystagmus observed in the right eye during abduction

**Figure 3:**
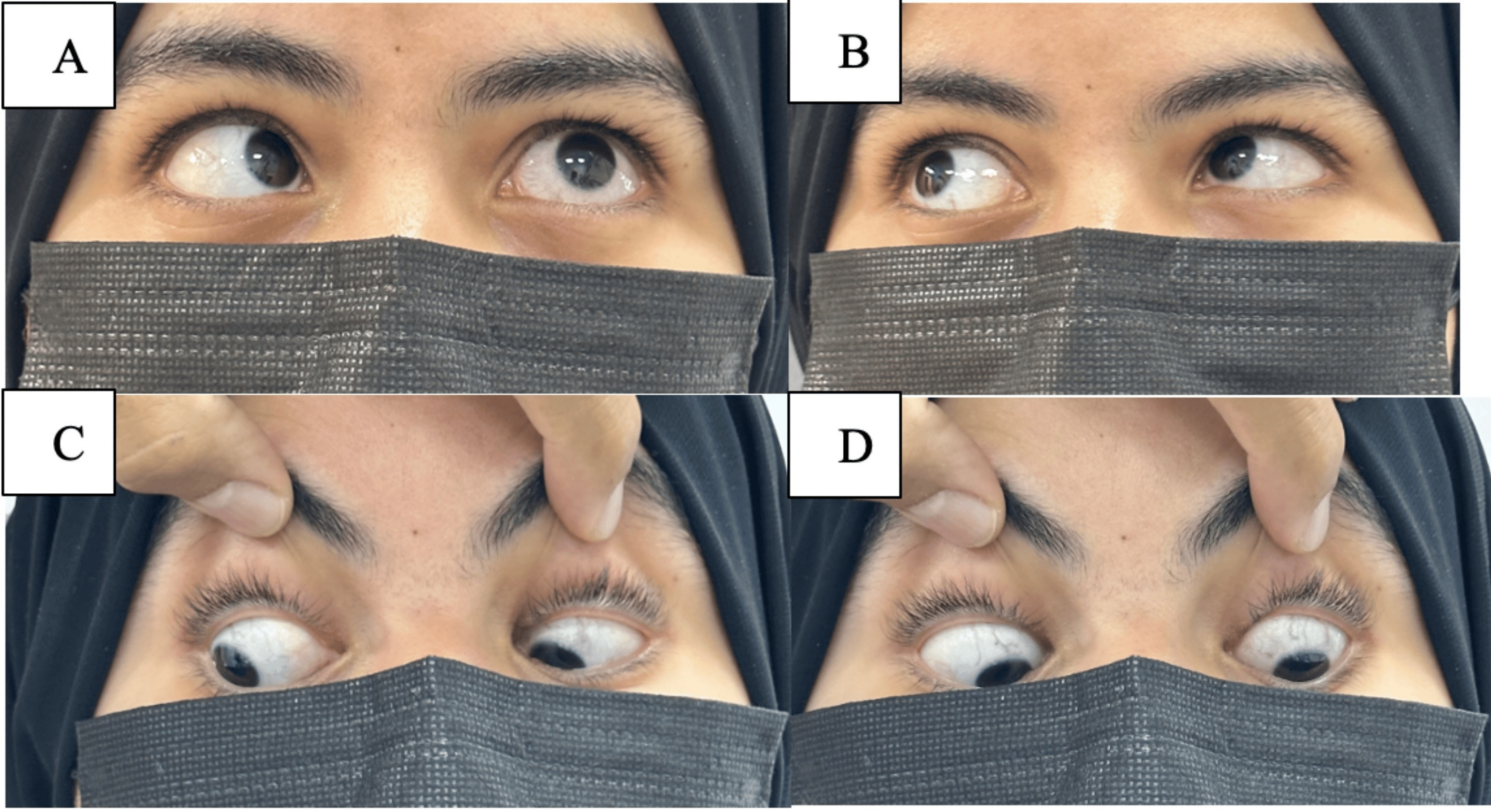
Oculomotor examination in various positions (A to D).

The rest of the motility exam was normal (Figure 3), and she was orthotropic in the primary position.

Slit-lamp examination details are mentioned in Table 1. 

**Table 1:** Slit-lamp examination findings of case 1 WNL: Within normal limit; RAPD: Relative afferent pupillary defect

Variable	Right eye (OD)	Left eye (OS)
Lid/lashes	WNL	WNL
Conjunctiva/sclera	WNL	Mild conjunctival injection
Cornea	Clear	Clear
Anterior chamber	Deep and quiet	Deep and quiet
Pupil	Regular, round, reactive, and no RAPD	Regular, round, reactive, and no RAPD.
Lens	Clear	Clear
Fundus	Clear view, flat retina, healthy disc and macula	Clear view, flat retina, healthy disc and macula
Auto-refraction	Sphere = 0.0 Cylinder = -0.5 x Axis = 75	Sphere = -0.75 Cylinder = 0.75 x Axis = 16

The clinical presentation aligned with Duane syndrome type 1, characterized by restricted abduction and narrowing of the palpebral fissure during adduction. Given her good vision, tolerance of head posture, and absence of significant symptoms, surgical intervention was deferred. The patient was scheduled for follow-up visits at intervals of six months to monitor for any changes in symptoms, vision, or head posture. At the six-month follow-up, the patient remained asymptomatic, with stable visual acuity and no significant changes in her motility findings.

Case 2

**Figure 4:**
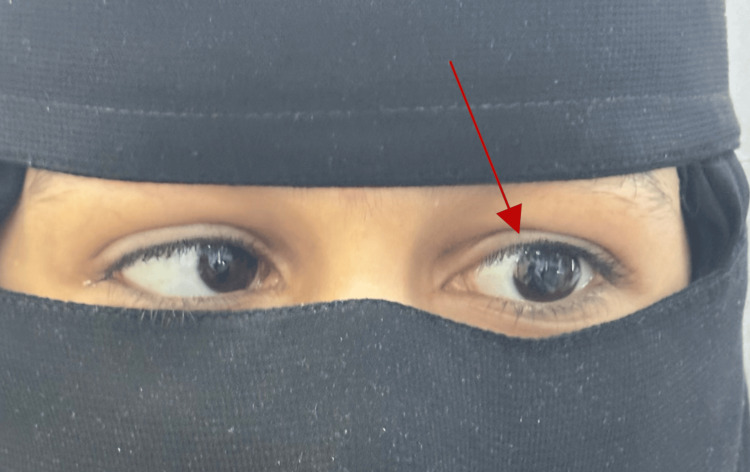
Deficit in left eye abduction

A 12-year-old female presented to the Ophthalmology Clinic with decreased vision in the left eye. She denied any sensation of double vision and denied any history of trauma, recent infections, or allergies. The patient had no significant past medical history and had not undergone any ocular surgeries. There were no known systemic illnesses. No relevant family history of ocular disorders, strabismus, or hereditary eye conditions existed. The systemic history was unremarkable, with no reports of headaches, nausea, or systemic symptoms such as fever or weight loss. Her visual acuity was 20/20 in the right eye and 20/50 in the left eye, as per an ophthalmological examination. On examination of ocular motility, there was a restriction of abduction in the left eye (Figure 4).

**Figure 5:**
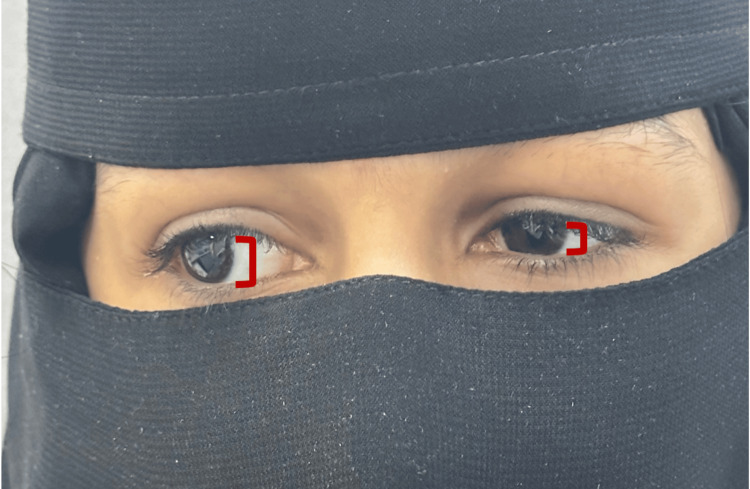
On adduction, the left eye narrows and retracts palpably

There was limited adduction with noticeable palpebral fissure narrowing on adduction, particularly on the left side (Figure 5).

**Figure 6:**
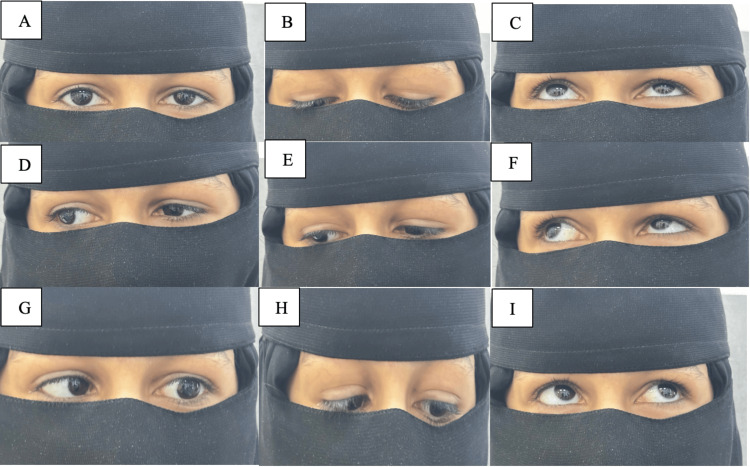
The remaining extraocular motility tests in each gaze (A to I)

Asymmetry in palpebral fissure height was noted during adduction. She was orthotropic in the primary position, with the rest of the motility examination showing no abnormalities in gaze or range of eye movement (Figure 6).

Slit-lamp examination details are mentioned in Table 2.

**Table 2:** Slit-lamp examination findings of case 2 WNL: Within normal limit; RAPD: Relative afferent pupillary defect

Variable	Right eye (OD)	Left eye (OS)
Lid/lashes	WNL	WNL
Conjunctiva/sclera	WNL	WNL
Cornea	Clear	Clear
Anterior chamber	Deep and quiet	Deep and quiet
Pupil	Regular, round, reactive, and no RAPD	Regular, round, reactive, and no RAPD
Lens	Clear	Clear
Fundus	Clear view, flat retina, healthy disc and macula	Clear view, flat retina, healthy disc and macula
Auto-refraction	Sphere = +5.25 Cylinder = -1.00 Axis = 156	Sphere = +6.00 Cylinder = -1.50 Axis = 155

An MRI was performed and yielded unremarkable findings. The clinical features were consistent with Duane syndrome type 1, presenting as limited abduction and palpebral fissure narrowing during adduction. Considering her good vision, tolerance of head posture, and minimal symptoms, surgery was not indicated. The patient was scheduled for follow-up visits every four months to monitor for changes in visual acuity, motility, and the need for potential intervention. At her first four-month follow-up, her visual acuity and motility findings remained stable, and she continued to tolerate her head posture well.

## Discussion

DRS is more common in women (58%). It is usually unilateral but can be bilateral and mostly affects the left eye (59%) [5]. Only 10% of patients have a familial profile, with most cases of DRS being sporadic. There are known dominant and recessive types, and they are often bilateral [6]. They showed varying levels of expressiveness and penetration among dominant types [7]. Similarly, in our case report, the patients are females and have a unilateral abduction deficit in the left eye. 

Although Duane syndrome is often an independent phenomenon, it can sometimes be associated with other abnormalities, including hearing loss, dysmorphic facial features, and renal, cardiac, and spinal anomalies. In rare cases, DRS can be associated with vesicoureteral reflux, Hirschsprung's disease, segmental focal glomerulonephritis, and genitourinary anomalies. Other syndromes including Goldenhar, Okihiro, Holtoram, Wildervanck, and Mobius syndromes can also be linked to it [8]. We are aware that both hereditary and environmental variables are involved; this most likely happens between weeks four and eight of pregnancy and is caused by the underdevelopment of specific brain stem regions that regulate the ocular muscles [7]. In our case report, the patients have only ocular signs and symptoms and have no associated systemic anomalies. Family history regarding this disease was also negative.

The oculomotor nerve abnormally innervates the lateral rectus muscle in Duane syndrome, and hypoplastic nuclei impair the correct development of the sixth cranial nerve that governs the muscle. The medial and lateral rectus muscles contract simultaneously causing retraction of the globe on adduction [8]. Likewise in our cases, the patients have an abduction deficit in the left eye and a narrowing of palpebral fissure on adduction.

Unlike abducent nerve palsy, since the oculomotor nerve innervates the lateral rectus muscle, DRS does not manifest as muscular atrophy. Conversely, additional anomalies include fibrosed muscles (15%), hypertrophied muscles (33%), and both fibrosed and hypertrophied muscles (11%). We also discovered that other muscles affected are the superior oblique, which hypertrophies in 7% of cases, and the medial rectus, which hypertrophies in 56% of cases [9]. Huber's famous classification divided DRS into three categories [10]. By examining abnormalities of the abducent nerve, MRI also helps to clarify clinical manifestations [11]. The most common type DRS type 1 is often confused with abducent nerve palsy. The sixth nerve's absence or hypoplasia and the fact that maximal innervation only reaches the lateral rectus muscle when the afflicted eye is abducted account for the apparent limiting of abduction [8]. In our cases, there is also an abduction deficit with limited adduction in the left eye and retraction of the globe that correlates with hypoplastic sixth nerve nuclei and anomalous innervation by the third nerve. Type 2 (7%) is an adduction limitation characterized by partial agenesis of the sixth nerve that does not compromise function [6,7]. Type 3 (15%) is characterized by co-contraction and lack of innervation of the lateral rectus muscle during an abduction attempt, which can be used to explain the limitations of adduction and abduction [7]. Our patients were diagnosed with type 1 DRS.

A summary of the literature on cases of Duane syndrome published since 2000, highlighting key findings is presented in Table 3.

**Table 3:** Summary of reported cases of Duane syndrome in the literature from 2000 to present including patient demographics and clinical features DRS: Duane retraction syndrome; BCVA: Best corrected visual acuity

Author/year	Patient demographic	Classic presentation	Examination findings	Type of syndrome
García et al., 2015 [12]	5-year-old boy	From birth, difficulty moving both eyes horizontally	Each eye's Snellen visual acuity was determined to be +1.5 D with spherical correction. When the palpebral fissure narrowed and the globe retracted somewhat, the extrinsic ocular motility test showed a significant restriction of adduction and an inability to abduct both eyes. Depending on the fixed eye, the patient's face turned to the left or right due to changeable moderate horizontal torticollis. The patient had alternating fixation without vertical changes and esotropia up to +20 PD in the principal location of gaze.	Bilateral type III Duane syndrome
Gökçe et al., 2019 [13]	1.5-year-old boy	Showed up with an odd head posture and strabismus in his left eye	In the primary gazing posture, his chin was angled upward and to the left, and his neck was angled to the right. There was no refractive error in either eye, and the right eye's gazes in all directions were normal. In the left eye, there was an abduction restriction. The predominant gaze position did not exhibit noticeable esophoria.	DRS type 1
Murthy et al., 2022 [14]	13‑year‑old boy	There was a noticeable narrowing of the left eye when facing the left	Both eyes had a BCVA of 6/6; N6. In adjusted head position, 40 arc-second stereopsis was seen on the Titmus fly test. He had a 15° left-face turn. In the main position, the alternate prism cover tests revealed exophoria, which progressed to 12 PD exotropia, with 7 PD hypertropia in the left gaze and orthophoria in the right. The right eye's ocular movements revealed a -2 grade adduction restriction, but the left eye was normal. Along with globe retraction in the right eye, a narrowing of the palpebral fissure was seen (palpebral fissure height: 9.5 mm on abduction and 13.5 mm on adduction). The results of cycloplegic refraction showed that the right eye had +0.50 DS and the left eye had +0.50 DS with -0.50 DC.	Inverse DRS
Mrad et al., 2024 [15]	13-year-old girl	At 21 months of life, left eye abducens paralysis was identified	She had a little left-head tilt, but her visual acuity was 20/20 in both eyes. The right eye's motility testing was normal. At the same time, it showed a significant retraction of the eyeball in adduction together with a restriction of abduction of the left eye. She was orthotropic in the primary position, and the remainder of the motility test was normal.	DRS type 1

Nystagmus is the most common eye condition that coexists with DRS. Ptosis, anisocoria, and epi-bulbar dermoid can also be observed in these patients. Furthermore, the literature has documented cases of Marcus-Gunn jaw-winking syndrome, heterochromia, optic nerve hypoplasia, and congenital cataracts. Patients with DRS may also have amblyopia and other refractive abnormalities like anisometropia [16,17]. In our patients, there was mild anisometropia but no ptosis, and fundus examinations were also unremarkable. Our patients had end gaze nystagmus in the right eye on adduction of the left eye [18].

Management options include observation and surgery. If abnormal head position and apparent retraction with adduction are present, surgical options may be chosen. However, any patient with normal vision in both eyes does not need treatment if there is no change in head position [16]. However, in cases where there is strabismus or head tilt, specialized treatment may be necessary. This may include correction of refractive error, amblyopia therapy, surgical techniques such as vertical muscle transposition or horizontal muscle recession, or a combination of these [15]. Any of the following characteristics should be considered whether to proceed with surgery on a patient: an abnormal head position greater than or equal to 15 degrees, a significant deviation from the primary position, and severely induced ptosis - a reduction greater than or equal to 50% in the height of the palpebral fissure after adduction [19]. As our patients were diagnosed with DRS type 1 with normal head posture, they are under observation, and we may proceed with surgery if a greater than 50% reduction in palpebral fissure is noted in the future.

## Conclusions

This study highlights the importance of awareness and timely recognition of DRS among healthcare professionals, particularly neurologists. Despite its rarity, DRS can be misdiagnosed or overlooked, leading to late diagnoses in individuals who may present with symptoms in adolescence or adulthood. Understanding the complexities of this condition is essential for appropriate management strategies, especially when patients demonstrate acceptable visual function. While surgical intervention may not be curative, effective conservative management can significantly enhance the quality of life by addressing compensatory head postures. Continued education and collaboration among pediatricians, ophthalmologists, and neurologists are crucial to improving outcomes for patients with DRS.
